# The Effect of Gentle Handling on Depressive-Like Behavior in Adult Male Mice: Considerations for Human and Rodent Interactions in the Laboratory

**DOI:** 10.1155/2018/2976014

**Published:** 2018-03-06

**Authors:** Caroline Neely, Christina Lane, Julio Torres, Jane Flinn

**Affiliations:** ^1^Department of Psychology, George Mason University, 4400 University Drive, Fairfax, VA 22030, USA; ^2^Interdisciplinary Program in Neuroscience, George Mason University, 4400 University Drive, Fairfax, VA 22030, USA

## Abstract

Environmental factors play a significant role in well-being of laboratory animals. Regulations and guidelines recommend, if not require, that stressors such as bright lighting, smells, and noises are eliminated or reduced to maximize animal well-being. A factor that is often overlooked is handling and how researchers interact with their animals. Researchers, lab assistants, and husbandry staff in animal facilities may use inconsistent handling methods when interacting with rodents, but humans should be considered a part of the animal's social environment. This study examined the effects of different handling techniques on depressive-like behavior, measured by the Porsolt forced swim test, in adult C57BL/6J male mice. The same two researchers handled the mice in a gentle, aggressive, or minimal (control) fashion over approximately two weeks prior to testing. The results demonstrated a beneficial effect of gentle handling: gentle handling reduced swimming immobility in the forced swim test compared to mice that were aggressively or minimally handled. We argue that gentle handling, rather than methodical handling, can foster a better relationship between the handlers and rodents. Although handling is not standardized across labs, consistent gentle handling allows for less challenging behavioral testing, better data collection, and overall improved animal welfare.

## 1. Introduction

Environmental conditions within an animal facility have great impact on the well-being of rodents used in behavioral experiments and can affect results, especially of tests that measure spontaneous behavior [1]. Regulations at animal facilities are dependent on certifications, institutional requirements, and the needs of individual principal investigators; however, most facilities follow recommendations, such as *The Guide* [2], that enforce ethical, valid, and scientific practices. Measures are taken in order to reduce or eliminate salient, unpleasant factors such as bright lighting [3–6], smells [1, 7–9], and loud noises [10] which negatively affect animal health.

An additional factor that may be considered a random implementation is handling prior to and/or during behavioral testing. Rodents are regarded as social animals [11–13], and social housing is recommended as standard care [2]. Care and handling become an integral part of an animal's routine [14, 15]; for example, rats can recognize humans and prefer to be with those that they know [16], but this has yet to be confirmed in mice. In light of these findings, personnel that perform care-related tasks and interact with laboratory animals should be considered a part of the animal's social experience. Lack of familiarity and relatedness among cagemates has been shown to induce stress [17] and aggression in mice, suggesting that unfamiliar experimenters may also provoke anxious, aggressive, or depressive-like states. Mice become less anxious and more receptive after multiple handling sessions by human caretakers but continue to show signs of escape [18]. What remains underreported is the description of quantity and quality of human-rodent social interactions [19], and how differences in handling affect animal's reactivity to human caretakers and overall behavior.

Lack of description of handling sessions may introduce experimenter error and bias [20]. For example, some experimenters may handle rodents in a gentle manner by permitting freedom of movement in the experimenter's hands or by preventing short-term tail suspension during cage changes. Criteria for what is considered gentle handling can vary from playful tickling of the rodent's back and stomach [21–23] to tapping or moving the homecage to maintain a state of arousal [24–26]. Using a playful handling technique, Costa et al. [23] demonstrated that this method reduced anxiety, improved cognitive behavior, and decreased norepinephrine secretion in rats. This study, however, measured the anxiolytic effects through increased entries into the open arm of an elevated plus maze. Other work has suggested that handling can be used as a substitute for social enrichment for animals that are individually housed due to aggression and/or illness. Cloutier and colleagues [21] demonstrated that social contact in the form of rough-and-tumble play for 2 minutes each day for 21 days reduced anxiety-related behavior and increased positive affective ultrasonic vocalizations of individually housed rats. This effect was also observed when researchers implemented the same rough-and-tumble play (through “tickling”) in juvenile rats, which were then injected intraperitoneally with saline 40 to 50 days after play. Handling before and after injections, and especially when implemented during juvenile development, reduced stress calls during injections and increased positive affective ultrasonic vocalizations [22]. Other research groups may handle in a minimally or less gently fashion, and this may explain discrepancies often found in published literature concerning rodent models of anxiety or depression [18, 27–29]. These findings further strengthen the notion that handling procedures need to be standardized, if not reported in published literature, in order to reduce confounds in affective measures.

Anxiety and depression are considered to have relatively high comorbidity in both humans and animals [30–32]; thus, the same handling techniques that decrease anxiety in laboratory animals may also decrease depression. Handling can be short-term, beginning several days prior to or during behavioral testing, or it may begin during neonatal development. Neonatal handling increases maternal behavior in the form of increased licking and grooming of pups, which in turn reduces behavioral fearfulness in offspring postweaning [33–36]. What remains uncertain is whether or not handling beginning in late adulthood and lasting for several days, rather than continuous handling for several weeks/months beginning after weaning, can reduce depression and mimic the same effects observed in neonatal-handling studies. In this experiment, we operationally defined gentle, aggressive, and minimal handling and examined the different handling styles on depressive-like behavior in adult mice by using the forced swim test (FST). The FST is used to measure depressive behaviors in rodents [37]; in addition, it is used to assess manipulations in experimental procedures with the intention of curbing or altogether avoiding depressive states [38]. We conducted the FST in order to examine if aggressive handling caused depressive-like behavior and/or if gentle handling improved an animal's resiliency. We hypothesized that gentle handling of mice would increase latency before immobility and would decrease duration of immobility in the FST compared to the control group. We also hypothesized that aggressive handling would decrease latency before immobility and increase duration of immobility when compared to the control group. This study examined the importance of rodent-human social interactions which can impact an animal's performance across a broad spectrum of behaviors.

## 2. Methods

All procedures were approved by the George Mason University Institutional Animal Care and Use Committee and were carried out in accordance with the National Institutes of Health Guide for the Care and Use of Laboratory Animals.

### 2.1. Animals

Twenty-one adult 7.5-month-old (*N* = 8) and 10-month-old (*N* = 13) C57BL/6J male mice (Jackson Laboratories, Bar Harbor, ME) were provided for the study. These mice were excess mice donated from other projects within the testing facility, were raised in homecages for respective periods of time prior to testing, and were naïve to handling and other testing procedures for approximately 3 months. Animals were group-housed by age. Homecages were lined with TEKFresh bedding (Envigo, Indianapolis, IN) and contained a PVC pipe and nylabone as additional forms of enrichment. The colony was maintained on a 12-hour light/dark cycle (lights on at 8:00 a.m.) with unautoclaved 7012 diet feed (Envigo, Indianapolis, IN) and water offered ad libitum. Cages were washed and autoclaved once a week by husbandry staff.

### 2.2. Handling

Mice were separated into three groups: a gently handled condition, an aggressively handled condition, and a control condition (Table 1). Mice were handled in the housing room under a lit biosafety cabinet by the same two experimenters (C.L. and J.T.). The mice were handled over the course of 13 days, every other day, in the evening hours during the light cycle (12:00–8:00 p.m.). The gently handled and aggressively handled groups were handled for 90 seconds per session, whereas the control group was not handled by the researchers. The gently handled mice were individually removed from their homecages, placed in the palm of the experimenter's hand, and were stroked on the left and right flanks and head for 90 seconds. Researchers carefully but firmly held the base of the tail, permitting movement between their hands. The aggressively handled mice were individually removed from their homecages, grasped at the proximal end of their tails, and suspended in the air approximately 15 cm from the surface of a biosafety cabinet for 90 seconds. Care was taken so that the mice would not latch onto any surrounding equipment or attempt to grasp the experimenter's fingers. The control mice were handled only by husbandry staff during routine cage changes; in addition, the gently and aggressively handled mice were handled by husbandry staff during this time.

### 2.3. Porsolt Forced Swim Test (FST)

Mice were individually transported to the testing room where they habituated for 10 minutes prior to testing. Three mice were tested at a time and were separated by an opaque barrier to obstruct viewing of other mice. Mice were removed from their transport cages by the base of their tail and slowly placed into an inescapable transparent Plexiglas cylinder (47 × 38 cm) filled to a depth of 32 cm with tap water (25–27°C). Mice were placed and released into the water at the same time. Each mouse was given a single 6-minute trial. Researchers manually recorded latency until the first period of immobility (seconds) and total duration immobile (seconds). Immobility was defined as the period of time that the mouse was not swimming, when movement was only made in order to keep the body in balance or its head above water [30]. At the end of the 6-minute trial, mice were removed from the cylinders and carefully dried and returned to their transport cages under a heat lamp positioned approximately 30 cm above the cage. Water was changed, and temperature was taken for the next trial. All mice successfully completed the 6-minute trial.

### 2.4. Statistical Analysis

Data were analyzed using R version 3.3.1 [39]. Latency to first instance of immobility (seconds) and total duration of immobility (seconds) were subjected to Levene's test of equality of variances. Due to unequal, small sample sizes and violations of normality, data were subjected to the nonparametric Mann–Whitney-Wilcoxon rank sum test and Kruskal-Wallis *H* test using the *FSA* package [40]. Significant Kruskal-Wallis results were followed by Dunn-Bonferroni pairwise comparisons that adjusted for familywise type I error rate. Statistical results are reported as mean ranks. Data points were considered outliers if they were beyond 1.5 times the interquartile range. The results were considered statistically significant if *p* < 0.05 and marginally significant if *p* < 0.10. Original box plots with medians were constructed using *ggplot2* [41] and regraphed in Microsoft Excel (2016) for publication purposes.

## 3. Results

We first examined any potential differences between the 7.5-month-old mice (*N* = 8) and 10-month-old mice (*N* = 13). A Mann–Whitney-Wilcoxon test yielded no significant differences in duration of immobility, *W* = 64.50, *p* = 0.38, with mean rank durations of 12.56 for the 7.5-month-old mice and 10.04 seconds for the 10-month-old mice. Likewise, latency before immobility was not significantly different, *W* = 60.50, *p* = 0.56, with mean ranks of 12.06 for the 7.5-month-old mice and 10.35 seconds for the 10-month-old mice.

**Figure 1:**
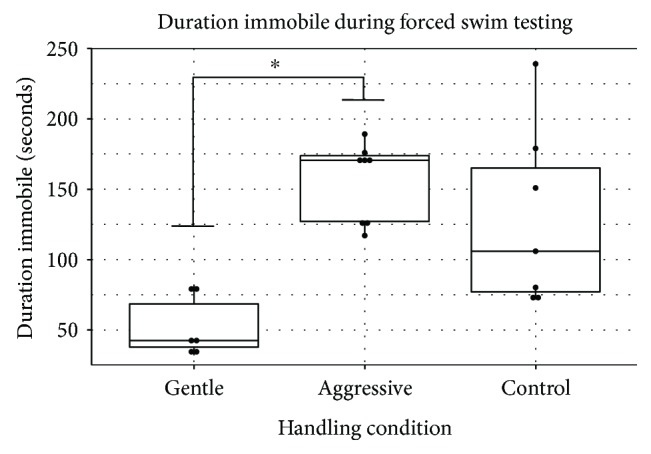
Duration immobile by handling group (gentle, aggressive, and control). The results demonstrate significant differences (∗) between the gently handled mice (median = 42.50 s) compared to the aggressively handled mice (median = 170.50 s) and marginally significant differences between the gently handled mice and control mice (median = 106.00 s). No differences were found between the aggressively handled and control mice. Box plots illustrate individual data points (solid dots), lower and upper quartiles (upper and lower sides of box plot), median (dark, middle bar), and lowest and highest values (whiskers).

**Figure 2:**
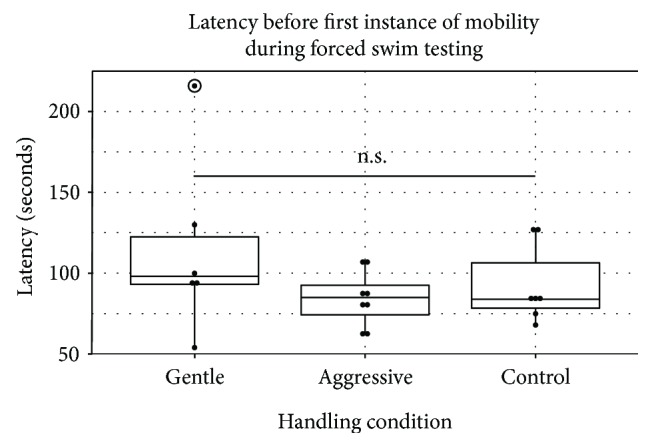
Latency prior to the first instance of immobility by the handling groups (gentle, aggressive, and control). There were no significant differences reported among conditions, with the gentle group showing the longest latency to immobility (median = 98.00 s), followed by the aggressive group (median = 85.00 s) and the control group (median = 84.00 s). Box plots illustrate the individual data points (solid dots), lower and upper quartiles (upper and lower sides of boxplot), median (dark, middle bar), and lowest and highest values (whiskers). An outlier is circled in the gently handled group.

We then examined the differences among the three handling conditions. A Kruskal-Wallis *H* test demonstrated a statistically significant difference in duration of immobility, *χ*^2^(2) = 10.80, *p* = 0.004, with a mean rank duration of 4.33, 15.25, and 11.86 seconds for the gentle, aggressive, and control groups, respectively (Figure 1). Pairwise comparisons showed a statistically significant difference between the gently (MR = 4.33 s) and aggressively handled (MR = 15.25 s) groups, *p* = 0.003, and marginally significant difference between the gentle and control (MR = 11.86 s) groups, *p* = 0.088. There were no statistical differences between the aggressive and control groups, *p* = 0.872. The proportion of variability for duration immobile accounted by the handling condition was approximately 54% (eta = 0.54), demonstrating a relatively strong effect of handling on depressive-like behaviors in the FST. Analyses on latency before immobility failed to yield statistically significant results, *χ*^2^(2) = 2.071, *p* = 0.355, with mean rank latencies of 14.00, 9.31, and 10.36 seconds for the gentle, aggressive, and control groups, respectively (Figure 2). The proportion of variability for latency accounted by the handling condition was approximately 10% (eta = 0.10). Overall, these data demonstrated that short-term, gentle handling decreases depressive-like behavior in the FST, in comparison to short-term, aggressive handling and handling by husbandry staff alone.

## 4. Discussion

The FST is a widely accepted method of examining the effectiveness of antidepressant pharmaceuticals and has been used to study other environmental manipulations. There is a large base of data measuring the effects of different pharmaceuticals on rodents and their performance in the FST [30]; however, the amount of data on the effects of handling on FST performance is not nearly as robust. We have demonstrated in this study that short-term gentle handling decreased depressive-like behavior in laboratory mice. The gently handled mice, as an overall group, demonstrated significantly less time immobile, indicating less depressive-like behavior. In comparison, the aggressively handled mice demonstrated the greatest duration immobile, indicating more depressive-like behavior. The control group, which was not handled except during routine husbandry procedures, performed intermediately compared to the gently and aggressively handled groups, showing greater duration immobile compared to the gently handled group. In addition, both methods of handling decreased variability in immobility in comparison to the control group (Figure 1). These data suggest that animals that had not been exposed to researchers prior to testing may react as if challenged by a mild stressor, or in the same way as if they have been aggressively handled.

Husbandry staff members were blind to handling conditions and handled all animals in the same fashion during routine cage changes; therefore, the lack of statistical difference between the aggressively handled and control groups cannot be accounted for by differences in husbandry staff's techniques. Exposure to husbandry staff, rather than exposure to the researcher performing testing, may not suffice for habituation to a human, especially if research concerns behavioral testing and affective-based measures. These results confirm that the way an animal is handled prior to or during behavioral testing can alter affective states. In addition, different handling techniques may be used for different testing procedures: gentle handling may provide a means of testing anxiolytic medications and the effects of stress, whereas aggressive handling may be a better method to detect the effects of antidepressant medications.

Handling has been extensively studied in younger animals, when the developing brain is susceptible to environmental stressors. It is understood that environmental enrichment and juvenile handling produces long-term neurobehavioral effects such as reduced anxiety [18] and improved spatial memory [42]. Handling, especially when implemented in early life and continued through adulthood, acclimatized rodents to human researchers and testing apparatuses. What is not extensively studied is whether handling in older mice can still mimic the benefits of prolonged handling seen in young, weaned mice. A challenge is that older mice may show age-related motor deficits. In this study, the older, 10-month-old mice swam longer (immobile for less time) compared to the 7.5-old mice but this difference was not significant (data not shown). Pooled analyses of 1800 C57BL/6J wild-type mice (independent of handling) showed that 6-7-month-old C57BL/6J mice swam longer than both 4-5-month and 2-3-month old mice in the forced swim test [43]. Other studies, however, have demonstrated an age-related degradation in general locomotion, strength, and endurance domains in mice [44] and swimming and sensitivity to antidepressant treatment in rats [45, 46] which could affect behavioral measures dependent on movement, such as the FST.

In this study, we were limited to the use of male mice, which were excess mice from breeding on other protocols that were kindly donated for behavioral testing. In neuroscientific research, there remains a strong sex bias with estimates suggesting that the use of male rodents in publications outnumber female rodents in a 5.5 to 1 ratio [47]. Research has emphasized that females are no more variable than males [48, 49], but the estrous cycle has been shown to affect responses to antidepressants [50–52]. A weakness of this experiment was that only males were included, which warrants the inclusion of female mice in future studies.

Research tends to favor handling-related behavioral changes in rats but not in mice. Handling in rats has been well studied possibly due to preference for the rat in experimental studies assessing learning and memory, beginning in the 1930s with the purposive behavioral work of Edward Tolman. Attention to mouse behavior has increased more recently due in large part to interest in transgenic mice [53, 54], with behavior often a secondary consideration. Rats have been said to be more sensitive than mice to issues of handling [55]; however, research has demonstrated that mice are also sensitive to types of handling and the presence of a researcher [56–58]. These findings, together with our research, further emphasize that mice should be handled prior to behavioral testing, and in such a way such that potential aggression towards cagemates and/or the researcher during testing does not impact the data obtained.

We handled over a period of two weeks with multiple sessions each week. Some studies do not report if animals were handled, the handling duration, and handling frequency. We note that our handling timeline is short term; however, a duration of two weeks may exceed what other labs consider adequate exposure to a researcher. A follow-up experiment from our lab examined how four consecutive days of gentle handling or husbandry only (i.e., control) handling prior to testing affected FST performance in 2-month-old mice. Although the gently handled mice displayed more resilient and less depressive-like behavior compared to the control group, these results were not significant (data not shown). Although there is no standard for handling, these data suggest that multiple handling sessions may be needed and that shorter time periods (e.g., 24 or 48 hours prior to testing) may not be suitable for habituating animals to researchers.

In conclusion, our study demonstrates the benefits of gentle handling of mice that undergo depression-based testing and shows that a gentle handling technique is effective when interacting with mice prior to and during behavioral testing. If implemented as the standard of care, handling can reduce the depressive symptoms in mice, improve the overall well-being of laboratory animals, produce more reliable data, and raise standards of care when using rodents as models for neuropsychiatric conditions.

## Figures and Tables

**Table 1:** Animal characteristics, by age and group.

Age	Gentle	Aggressive	Control
7.5 months	1	4	3
10 months	5	4	4
Total (*N* = 21)	6	8	7

## Data Availability

Raw data (.csv) and *FSA* and *ggplot2* R script (.R) are available upon request to the corresponding author. Please note that the R script was processed in RStudio and contains annotations from the corresponding author.
